# Endothelial cell-derived interleukin-6 regulates tumor growth

**DOI:** 10.1186/1471-2407-14-99

**Published:** 2014-02-17

**Authors:** Kathleen G Neiva, Kristy A Warner, Marcia S Campos, Zhaocheng Zhang, Juliana Moren, Theodora E Danciu, Jacques E Nör

**Affiliations:** 1 Angiogenesis Research Laboratory, Department of Cariology, Restorative Sciences, and Endodontics, University of Michigan School of Dentistry, Ann Arbor, Michigan 48109-1078, USA; 2 Department of Periodontics and Oral Medicine, University of Michigan School of Dentistry, Ann Arbor, Michigan 48109-1078, USA; 3 Department of Biomedical Engineering, University of Michigan College of Engineering, Ann Arbor, Michigan 48109-1078, USA; 4 Department of Otolaryngology, University of Michigan School of Medicine, Ann Arbor, Michigan 48109-1078, USA; 5 Comprehensive Cancer Center, University of Michigan, Ann Arbor, Michigan 48109-1078, USA; 6 Angiogenesis Research Laboratory, University of Michigan School of Dentistry, 1011 N. University Rm. 2309, Ann Arbor, MI 48109-1078, USA

**Keywords:** Cervical Cancer, Signaling pathways, Molecular targeted therapy, STAT3

## Abstract

**Background:**

Endothelial cells play a complex role in the pathobiology of cancer. This role is not limited to the making of blood vessels to allow for influx of oxygen and nutrients required for the high metabolic demands of tumor cells. Indeed, it has been recently shown that tumor-associated endothelial cells secrete molecules that enhance tumor cell survival and cancer stem cell self-renewal. The hypothesis underlying this work is that specific disruption of endothelial cell-initiated signaling inhibits tumor growth.

**Methods:**

Conditioned medium from primary human dermal microvascular endothelial cells (HDMEC) stably transduced with silencing RNA for IL-6 (or controls) was used to evaluate the role of endothelial-derived IL-6 on the activation of key signaling pathways in tumor cells. In addition, these endothelial cells were co-transplanted with tumor cells into immunodefficient mice to determine the impact of endothelial cell-derived IL-6 on tumor growth and angiogenesis.

**Results:**

We observed that tumor cells adjacent to blood vessels show strong phosphorylation of STAT3, a key mediator of tumor progression. In search for a possible mechanism for the activation of the STAT3 signaling pathway, we observed that silencing interleukin (IL)-6 in tumor-associated endothelial cells inhibited STAT3 phosphorylation in tumor cells. Notably, tumors vascularized with IL-6-silenced endothelial cells showed lower intratumoral microvessel density, lower tumor cell proliferation, and slower growth than tumors vascularized with control endothelial cells.

**Conclusions:**

Collectively, these results demonstrate that IL-6 secreted by endothelial cells enhance tumor growth, and suggest that cancer patients might benefit from targeted approaches that block signaling events initiated by endothelial cells.

## Background

Uterine cervix carcinoma (UCC) includes malignant lesions arising from the tissues of the cervix, and represents the 3^rd^ most common cancer in women worldwide with approximately 529,800 new cases diagnosed every year [1]. The three major histological types of invasive cervical cancer are squamous cell carcinoma (SCC), adenocarcinomas (AC) and adenosquamous carcinoma (ASC). SCC comprise 80% of cases, and adenocarcinomas and ASC comprise approximately 20% [1,2]. In developed countries, its incidence has showed a marked decline over the past 40 years because of widespread screening with cervical cytology. This decline is mainly attributable to a decrease in the incidence of squamous cell carcinoma [3-10]. On the other hand, there has been a relative increase in the incidence of adenocarcinomas and adenosquamous carcinoma of the cervix over the same period. Notably, the pathobiology of adenocarcinomas remains unclear, particularly the impact of the crosstalk between endothelial cells and tumor cells to cancer growth and progression. Better understanding of signaling events that mediate endothelial cell-tumor cell interactions will lead to the development of improved therapies for uterine cervix adenocarcinomas.

Tumor progression requires the formation of new blood vessels [11]. Therefore, several angiogenesis inhibitors have been developed to target endothelial cells and block tumor growth [12]. Targeting cells that support tumor growth, rather than the cancer cells themselves, is an attractive approach for cancer therapy. The vascular endothelium is directly accessible to drugs injected in the circulation, and is composed of cells that are more stable genetically when compared to cancer cells [13-15]. Notably, studies have suggested that both tumor and non-tumor cells may be involved in reduced responsiveness to therapy by developing acquired resistance [16]. Despite significant advances in therapies targeting angiogenic molecules, the survival benefits of these treatments are relatively modest [13], the treatments are costly [17], and have significant side effects [18,19]. In addition, single-agent therapy that is effective initially may ultimately lead to drug resistance [20] and tumor recurrence.

The development of molecular targeted therapies may lead to the rational selection of treatment for adenocarcinoma patients based on specific molecular mechanisms whose deregulated activity contributes to the initiation, development, and metastatic spread [21-24]. The deregulation of signaling cascades including the transcription factor signal transducer and activator transcription 3 (STAT3) pathway has been implicated in the pathogenesis of cervical cancer [21]. Notably, the overexpression of activated STAT3 is accompanied by poor prognosis in this sub-group of tumors [22]. It is well known that recombinant interleukin-6 (IL-6) induces STAT3 activation [23]. However, the effect of endothelial cell-secreted IL-6 on tumor cell STAT3 and overall tumor growth is not known. The characterization of the functional impact of the crosstalk between endothelial cells and tumor cells on tumor growth and progression may unveil endothelial cell-secreted molecules as a new conceptual target for cervical cancer therapy.

The prevalent paradigm in tumor biology is that tumor cells secrete factors that drive tumor growth and that endothelial cells simply respond by generating new blood vessels that support the high metabolic demands of tumor cells. Here, we challenged this paradigm and observed that endothelial cell IL-6 levels have a direct impact on tumor cell phenotype and tumor growth *in vivo*. Our results demonstrate that endothelial cell-secreted IL-6 defines the growth of adenocarcinomas in preclinical models.

## Methods

### Cell culture

Cervical adenocarcinoma cells (HeLa Cells) were cultured in Dulbecco’s Modified Eagle Medium (DMEM; Invitrogen, Carlsbad, CA) supplemented with 10% fetal bovine serum (FBS), 100 U/ml penicillin, and 100 μg/ml streptomycin (Invitrogen). Tumor cells were serum-starved overnight before adding treatment. An immortalized human oral keratinocyte cell line (HOK-16B, gift of No-Hee Park, University of California, Los Angeles) was cultured in serum free medium (OKM; ScienCell, Carlsbad, CA) containing 1% penicillin/streptomycin, and supplemented with 5 μg/ml BSA, 5 μg/ml transferring, 50 μg/ml bovine pituitary extract, 2.5 μg/ml insulin, 1 ng/ml FGF, 500 ng/ml epinephrine, 1 μg/ml hydrocortisone, 30 nM prostaglandin, and 40 μg/ml plant extract (OKGS, BulletKit, ScienCell). Primary human dermal microvascular endothelial cells (HDMEC; Cambrex, Walkersville, MD) were cultured in endothelial growth medium-2 (EGM2-MV; Cambrex). Conditioned medium (CM) from HDMEC or HeLa were prepared in endothelial cell medium (EBM) without supplementation with growth factors or serum from 24-hour cultures.

### Stable short hairpin RNA (shRNA) transduction

Lentiviruses expressing a short hairpin RNA (shRNA) construct for silencing IL-6 (Vector Core, University of Michigan) were generated in human embryonic kidney cells (293 T) transfected by the calcium phosphate method, as described [25]. A scrambled oligonucleotide sequence (shRNA-C) was used as control. Supernatants were collected 48 hours after transfection and used to infect HDMEC in 1:1 dilution medium containing 4 μg/ml polybrene (Sigma-Aldrich, St. Louis, MO). Cells were selected in EGM2-MV supplemented with 1 μg/ml puromycin (InvivoGen, San Diego, CA). Downregulation of IL-6 was confirmed by ELISA.

### Western blots

8 × 10^5^ HeLa were plated in 60 mm dishes, starved overnight, and exposed to EBM, or conditioned medium (CM) collected from HDMEC or HeLa for the indicated time points. HDMEC CM and HeLa CM were normalized by total protein concentration. In addition, HOK-16B were exposed to HDMEC CM. Alternatively, tumor cells were exposed to rhIL-6 (BDP, NCI, Frederick, MD) for the indicated time points. Signaling pathways were blocked by pre-incubating tumor cells for 1-2 hours with 20 μM Stattic (STAT3 inhibitor V, Calbiochem, San Diego, CA), 20 μM LY294002 (PI3 kinase inhibitor, Cell Signaling Technology, Danvers, MA), or 20 μM U0126 (MEK1/2 inhibitor, Cell Signaling), as described [26], and exposed to HDMEC CM or rhIL-6 for the indicated time points. Lysates (30 μg) were electrophoresed in SDS-polyacrylamide gels and transferred to nitrocellulose membranes. Primary antibodies were: mouse anti-human phospho-STAT3, rabbit anti-human STAT3, rabbit anti-human phospho-Akt, rabbit anti-human Akt, rabbit anti-human phospho-ERK1/2, mouse anti-human ERK1/2 (Cell Signaling); and mouse anti-glyceraldehyde-3-phosphate dehydrogenase (GAPDH; Chemicon, Millipore, Billerca, MA). Phosphorylation antibodies detected endogenous levels of STAT3, Akt, and ERK1/2 when phosphorylated at Tyrosine 705, Serine 473, and Threonine 202/Tyrosine 204, respectively. Immunoreactive proteins were visualized by SuperSignal West Pico chemiluminescent substrate (Thermo Scientific, Rockford, IL).

### Enzyme-linked immunosorbant assay (ELISA)

Supernatants of endothelial or tumor cell cultures (24 hours) were collected and centrifuged. IL-6 expression was determined using ELISA kits (Quantikine; R & D Systems, Minneapolis, MN) according to the manufacturer’s instructions. Data were normalized by cell number.

### SCID mouse model of human tumor angiogenesis

Xenograft human tumors vascularized with human blood vessels were generated under an UCUCA approved protocol, as described [27-29]. Briefly, highly porous poly-L(lactic) acid (Boehringer Ingelheim, Ingelheim, Germany) scaffolds were seeded with 9 × 10^5^ HDMEC and 1 × 10^5^ HeLa in a 1:1 mixture of growth factor reduced Matrigel and EGM2-MV. In addition, scaffolds were seeded with 9 × 10^5^ HDMEC-shRNA-control or HDMEC-shRNA-IL-6 and 1 × 10^5^ HeLa. Severe combined immunodeficient (SCID) mice (5-7-week-old male CB.17.SCID; Charles River, Wilmington, MA) were anesthetized with ketamine and xylazine, and 2 scaffolds were implanted in the subcutaneous space of the dorsal region of each mouse, *i.e.* one scaffold seeded with HDMEC-shRNA-control + HeLa and one scaffold seeded with HDMEC-shRNA-IL-6 + HeLa. Tumors were measured with a caliper every 2 days, starting at 14 days after implantation. Mice were euthanized after 28 days, implants were retrieved, photographed, measured, weighed, fixed overnight in 10% buffered formalin at 4°C, and embedded in paraffin following standard histological procedures. These studies were performed two independent times to verify the reproducibility of the work under a protocol reviewed and approved by the University of Michigan Committee on Use and Care of Animals (UCUCA). The total “n” of each experimental condition was n = 12 tumors.

### Immunohistochemistry of tissue sections

Immunohistochemistry was performed in paraffin-embedded serial sections using phospho-STAT3 (Santa Cruz), STAT3, phospho-Akt, Akt, phospho-ERK, ERK (Cell Signaling), and Ki67 (Biocare Medical, Concord, CA) antibodies, as described [30].

### Tumor microvessel density

Tumor microvessel density was determined following identification of blood vessels by immunohistochemistry with a polyclonal anti-human factor VIII antibody (Lab Vision, Fremont, CA), as previously described [27,28]. The number of stained microvessels was counted in 10 random fields per implant in a light microscope at 100×. Twelve implants were analyzed per condition.

### Statistical analyses

T-tests or one-way ANOVA followed by appropriate post-hoc tests were performed using SigmaStat 2.0 (SPSS; Chicago, IL). Statistical significance was determined at P < 0.05.

## Results

### Endothelial cell-secreted factors activate key signaling pathways in tumor cells

**Figure 1:**
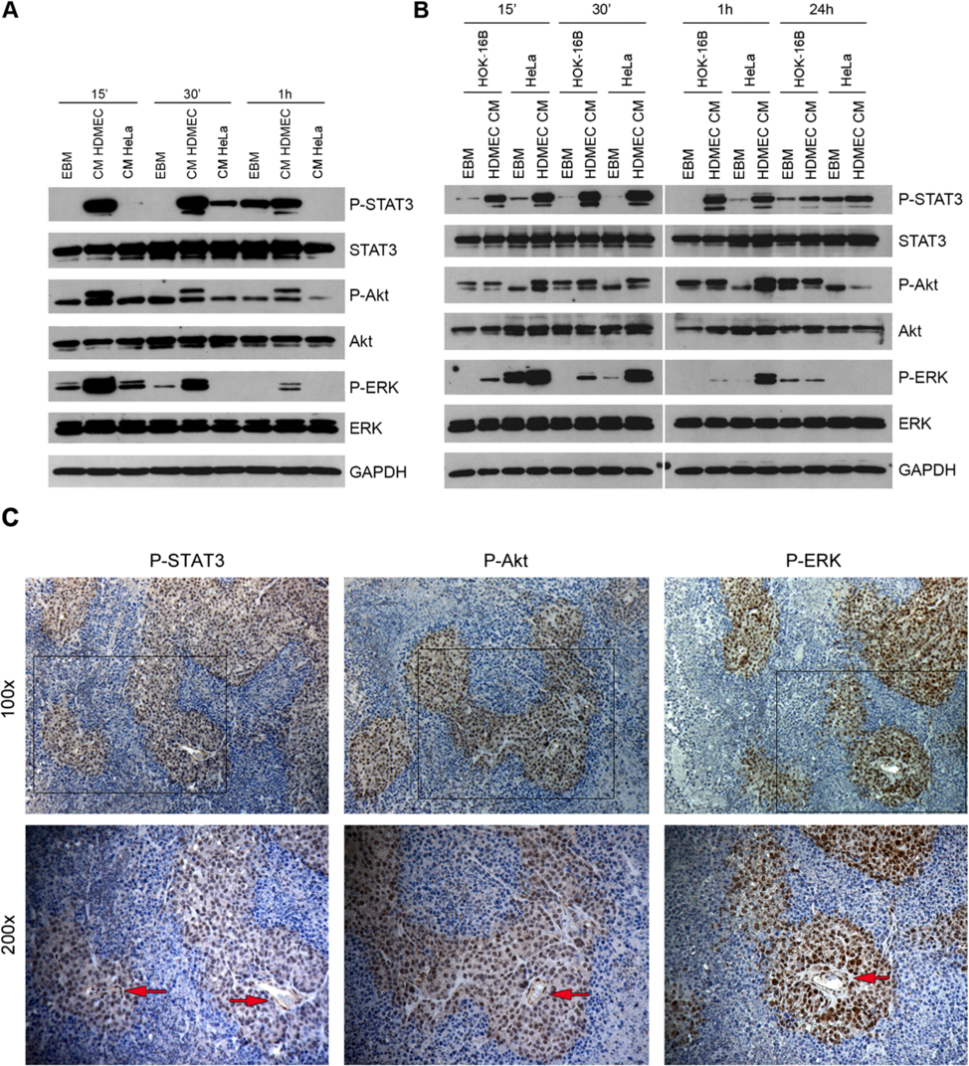
**Endothelial cell-derived factors phosphorylate STAT3, Akt, and ERK in tumor cells *in vitro *and *in vivo*. A**, western blot for phosphorylated and total STAT3, Akt, and ERK in HeLa serum-starved overnight and exposed to HDMEC conditioned medium (CM), HeLa CM, or control unconditioned medium (EBM) for the indicated time points. **B**, western blot for phosphorylated and total STAT3, Akt, and ERK in HeLa or HOK-16B serum-starved overnight and exposed to HDMEC CM or EBM for the indicated time points. **C**, immunohistochemical analysis for phosphorylated STAT3, Akt, and ERK (with nuclear localization) in representative specimens from xenograft human squamous cell carcinomas. Top panels represent 100× and lower panels represent 200× magnification. Red arrows point to blood vessels.

We have previously demonstrated that a crosstalk initiated by endothelial cells enhances tumor cell survival and migration *in vitro,* and that endothelial cell-derived IL-6 induces phosphorylation of STAT3 in tumor cells [26]. The overall hypothesis underlying this study is that the activation of signaling pathways in tumor cells induced by endothelial cell-secreted factors enhances tumor growth. To begin to address this hypothesis, we exposed HeLa cells to serum-free endothelial cell (HDMEC) conditioned medium (CM) or tumor cell (HeLa) CM and analyzed phosphorylation events over time (Figure 1A). We observed that phosphorylation levels of STAT3, Akt, and ERK were higher in tumor cells exposed to HDMEC CM than in tumor cells exposed to HeLa CM, or unconditioned medium (EBM). The induction of phosphorylation was observed primarily at early time points (15 to 30 minutes), decreasing at 1 hour (Figure 1A). Notably, expression levels of IL-6 were higher in HDMEC CM than in HeLa CM, and silencing IL-6 in endothelial cells did not have a measurable impact in endothelial cell proliferation (data not shown). In addition, we analyzed phosphorylation events on HeLa cells and on keratinocytes (HOK-16B) exposed to HDMEC CM or unconditioned medium (EBM) (Figure 1B). We observed that phosphorylation levels of STAT3, Akt, and ERK were higher when both tumor cells and keratinocytes were exposed to HDMEC CM than to EBM. Similarly, phosphorylation was observed mainly at early time points and decreased at 24 hours (Figure 1B).

To evaluate whether the trends of endothelial cell-induced phosphorylation of STAT3, Akt, and ERK in tumor cells *in vitro* translate into increased phosphorylation levels *in vivo*, we used the SCID mouse model of human tumor angiogenesis in which we engineer cervical cell adenocarcinomas vascularized with human functional blood vessels that anastomize with the mouse vasculature [27-29]. We implanted highly porous biodegradable scaffolds containing primary human endothelial cells (HDMEC) together with cervical adenocarcinoma cells (HeLa) in the subcutaneous of SCID mice and analyzed the tissues by immunohistochemistry 28 days after transplantation. We observed that tumor cells adjacent to blood vessels showed phosphorylation of STAT3, Akt, and ERK (Figure 1C). In contrast, the expression of total STAT3, Akt, and ERK was relatively uniform throughout the tissues (data not shown).

### Endothelial cell-induced STAT3 phosphorylation is independent of Akt and ERK

**Figure 2:**
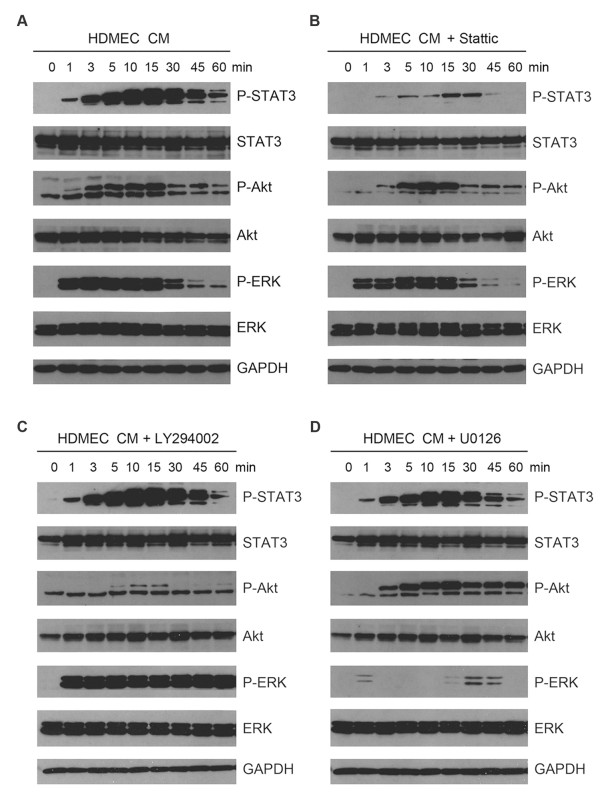
**STAT3 phosphorylation induced by endothelial cell-secreted factors is independent of Akt and ERK phosphorylation.** Western blot for phosphorylated and total STAT3, Akt, and ERK in HeLa serum-starved overnight and exposed to **A**, HDMEC conditioned medium (CM) or unconditioned medium (EBM) for the indicated time points. In addition, HeLa were pre-incubated for 1 to 2 hours with **B**, 20 μM Stattic; **C**, 20 μM LY294002; or **D**, 20 μM U0126, and then exposed to HDMEC CM or EBM in presence of the specific inhibitor for the indicated time points.

To explore the interdependence of molecular signaling events initiated by endothelial cells on tumor cells, we exposed HeLa to HDMEC CM in the presence of chemical inhibitors of STAT3, Akt, or ERK pathways and analyzed the interdependency of the phosphorylation events. To establish the baseline for these experiments, we exposed HeLa to HDMEC CM and analyzed phosphorylation of STAT3, Akt, and ERK with a detailed time course up to 1 hour (Figure 2A). We observed that HDMEC CM induces first ERK phosphorylation (with strong activation as early as 1 minute, persisting until 15 minutes, and decreasing at 30 minutes), followed by STAT3 and Akt (increasing until 15 minutes, and maintaining activation for up to 1 hour) (Figure 2A). When we inhibited STAT3 phosphorylation using the chemical inhibitor Stattic, we did not observe significant changes in phosphorylation of Akt or ERK (Figure 2B). However, when we inhibited Akt phosphorylation using the PI3K inhibitor LY294002 we observed an increase in ERK phosphorylation levels (maintaining strong phosphorylation for up to 1 hour), while phosphorylation levels of STAT3 did not change (Figure 2C). Similarly, when we inhibited ERK phosphorylation using the MEK1/2 inhibitor U0126 we observed increased Akt phosphorylation (maintaining strong phosphorylation for up to 1 hour), whereas phosphorylation levels of STAT3 remained unchanged (Figure 2D).

Then, we extended the time course experiments to 24 hours, and observed the same relationship between STAT3, Akt, and ERK phosphorylation in tumor cells induced by endothelial cell-secreted factors (Additional file 1: Figure S1). STAT3, Akt, and ERK phosphorylation were stronger at early time points (15 to 30 minutes), and decreased over time. STAT3 phosphorylation decreased at 1 hour and was maintained for up to 24 hours, phosphorylation of Akt decreased at 2 hours and disappeared at 4 to 24 hours, while phosphorylation of ERK decreased significantly at 1 hour and was absent at 3 to 24 hours (Additional file 1: Figure S1A). Inhibition of STAT3 phosphorylation did not affect Akt or ERK phosphorylation levels (Additional file 1: Figure S1B). On the other hand, inhibition of Akt phosphorylation increased activation of ERK (Additional file 1: Figure S1C), and inhibition of ERK phosphorylation increased Akt activation (Additional file 1: Figure S1D). No major effect was observed in STAT3 phosphorylation levels using Akt or ERK inhibitors. Collectively, these studies demonstrated that endothelial cell-induced Akt and ERK phosphorylation in tumor cells induce a mutually compensatory effect, while the STAT3 pathway is activated independently.

### IL-6 induces the STAT3 signaling pathway in tumor cells

**Figure 3:**
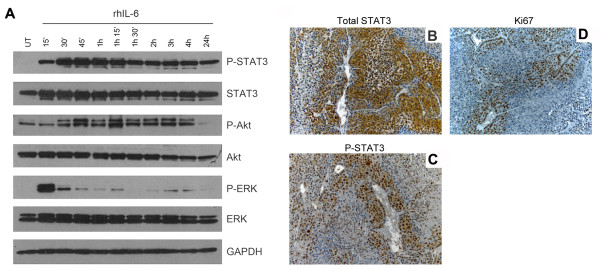
**STAT3 phosphorylation in xenograft human oral squamous cell carcinomas correlates with tumor cell proliferation and presence of blood vessels. A**, HeLa cells were serum-starved overnight and exposed to 20 ng/ml rhIL-6 for the indicated time points. Phosphorylated and total levels of STAT3, Akt, and ERK were determined by Western blots. **B-D**, xenograft human tumors were generated in SCID mice by co-implanting HeLa and HDMEC. Tumors were retrieved after 28 days, and tissues were analyzed by immunohistochemistry: **B**, total STAT3 with cytoplasmic localization, diffused through the tissue; **C**, phosphorylated STAT3 with nuclear localization, concentrated in the proximity of blood vessels; **D**, Ki67 with nuclear translocation, localized primarily around blood vessels. Photomicrographs at 200×.

Considering the clinical relevance of the STAT3 signaling pathway in cervical carcinoma [21,22] we focused the remaining studies of this work on the effect of endothelial cell-secreted IL-6 in the biology of adenocarcinoma cells. To understand the Cervical Adenocarcinoma response to IL-6 stimulation, we performed a detailed time course analyzing the phosphorylation events in HeLa cells (Figure 3A). We observed that when tumor cells were exposed to rhIL-6, the phosphorylation of STAT3, Akt, and ERK followed similar patterns as when tumor cells were exposed to HDMEC CM (Figure 1A; Additional file 1: Figure 1A). We then exposed tumor cells to IL-6 in the presence of chemical inhibitors of STAT3, Akt, or ERK pathways and analyzed the phosphorylation responses (Additional file 1: Figure S2). IL-6 strongly activated STAT3 pathway in HeLa, and slightly activated Akt or ERK (Additional file 1: Figure S2A). Blockade of STAT3 phosphorylation had no major effect on Akt but increased ERK phosphorylation (Additional file 1: Figure S2B). Inhibition of Akt had no effect on STAT3, while increased ERK phosphorylation (Additional file 1: Figure S2C). Lastly, inhibition of ERK phosphorylation had no significant effect on STAT3 or Akt phosphorylation (Additional file 1: Figure S2D). Collectively, these results showed that IL-6 is a potent inducer STAT3 signaling, while it has a weaker effect on the phosphorylation of Akt and ERK in Cervical Adenocarcinoma.

These results led us to further explore the IL-6/STAT3 signaling *in vivo.* We used the SCID mouse model of human tumor angiogenesis to generate human adenocarcinomas. We observed that while total STAT3 was present diffusely through the entire tissue (Figure 3B, a), phosphorylated STAT3 showed a tendency to localize adjacent to blood vessels (Figure 3B, b). Interestingly, immunostaining for the cell proliferation marker Ki67 showed the same pattern as phosphorylated STAT3 (Figure 3B, c). These results suggested that phosphorylation of STAT3 in xenograft carcinomas correlates with tumor cell proliferation and the proximity to blood vessels.

### Silencing of endothelial cell-IL-6 is sufficient to inhibit tumor growth

**Figure 4:**
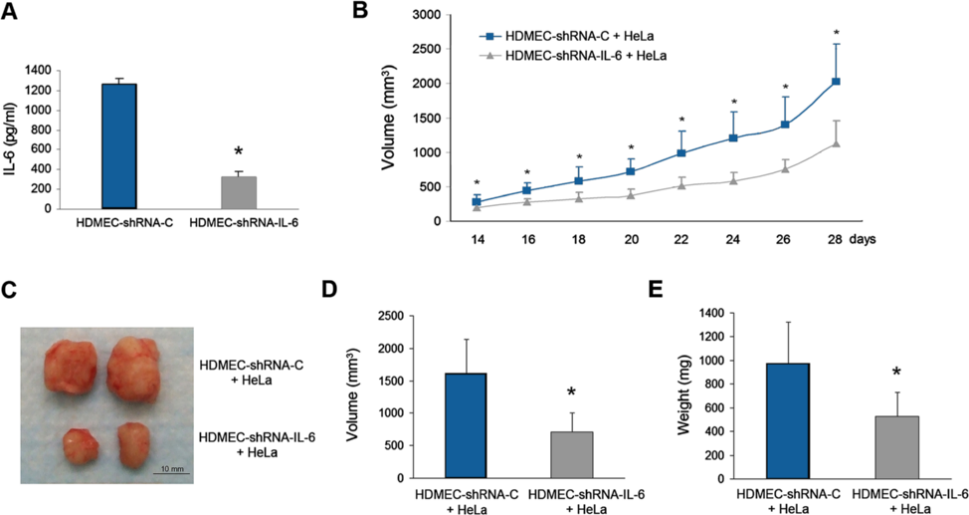
**Downregulation of IL-6 in tumor-associated endothelial cells inhibits tumor growth. A**, ELISA for IL-6 expression in HDMEC transfected with shRNA-IL-6 or with a control scrambled oligonucleotide sequence (shRNA-C). **B**, xenograft human tumors were generated in SCID mice by co-implanting HeLa and HDMEC-shRNA-IL-6 or control HDMEC-shRNA-C. Tumors growth was analyzed with calipers every 2 days for the duration of the experiment. **C**, macroscopic view of two representative xenograft tumors per group. **D-E**, graphs depicting tumor volume **(D)** and tumor weight **(E)** after retrieval (28 days post-implantation). Asterisk depicts p < 0.05.

To investigate whether these *in vitro* trends have a biological effect *in vivo*, we generated xenograft tumors vascularized with endothelial cells secreting low levels of IL-6 (HDMEC-shRNA-IL-6) or empty vector control endothelial cells (Figure 4A). Tumors populated with HDMEC-shRNA-control grew significantly faster and reached 2,000 mm^3^ at 28 days after implantation, whereas tumors vascularized with IL-6-silenced endothelial cells presented approximately half of this size (Figure 4B and C). Indeed, IL-6 silencing specifically in the vascular endothelial cells was sufficient to significantly slow down xenograft tumor growth (Figure 4B and C). Tumors populated with control endothelial cells also presented significantly higher volume (Figure 4D) and weight (Figure 4E) than tumors populated with IL-6-downregulated endothelial cells.

**Figure 5:**
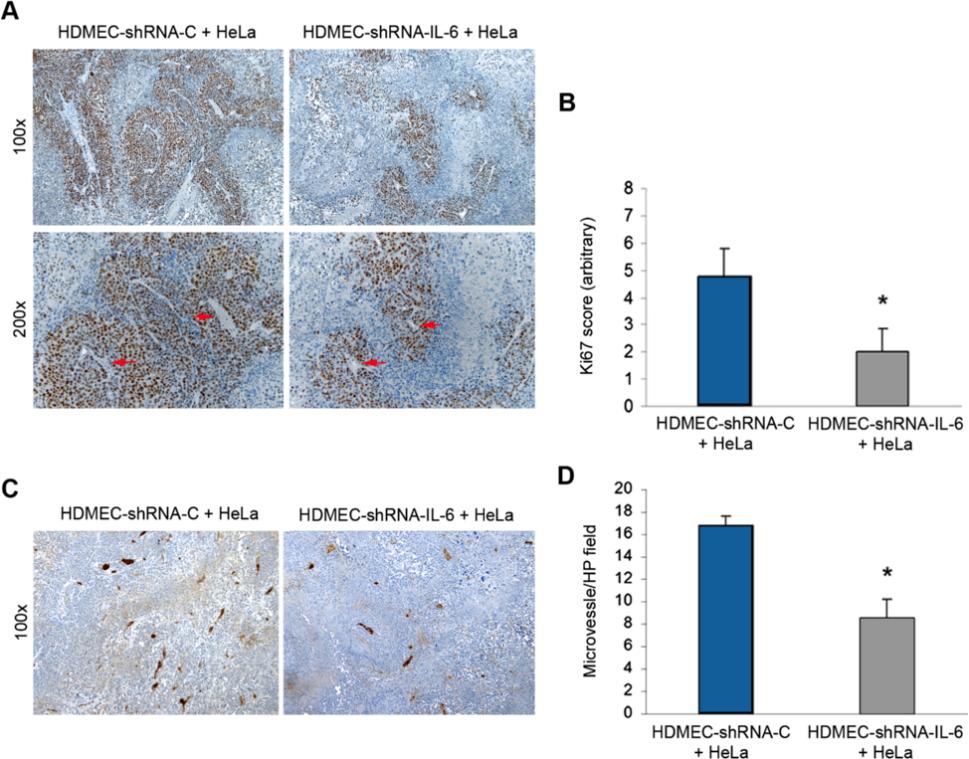
**Downregulation of IL-6 in tumor-associated endothelial cells reduces tumor cell proliferation and decreases intratumoral microvessel density. A**, immunohistochemical analysis for Ki67, indicating tumor cell proliferation in implants containing HeLa and HDMEC-shRNA-C, or HeLa and HDMEC-shRNA-IL-6. Top panels represent 100× and bottom panels represent 200×. **B**, quantification of tumor cell proliferation determined by scoring Ki67 immunostaining. Data represent mean values obtained in random microscopic fields (100×) from 12 tumors per condition. Asterisk depicts p < 0.05. **C**, immunohistochemistry for Factor VIII depicting blood vessels (100×). Left panel shows a representative tumor populated with HeLa and HDMEC-shRNA-C, and right panel shows a tumor containing HeLa and HDMEC-shRNA-IL-6. **D**, quantification of the microvessel density in these tumors. Data represent mean values obtained in 10 random microscopic fields per implant (100×) from 12 tumors per condition. Asterisk depicts p < 0.05. Red arrows point to blood vessels.

To explore the mechanisms involved in the inhibition of tumor growth mediated by the silencing of endothelial cell-IL-6, we analyzed tumor cell proliferation and intratumoral microvessel density by immunohistochemistry. We observed that expression of the proliferation marker Ki67 was lower in tumors cells when xenografts were vascularized with IL-6-silenced endothelial cells (Figure 5A and B). We also observed a decrease in microvessel density in tumors vascularized with endothelial cells with downregulated IL-6 expression, as compared to xenografts vascularized with control endothelial cells (Figure 5C and D). Taken together, these results demonstrated that downregulation of IL-6 in tumor-associated endothelial cells is sufficient to inhibit tumor growth.

## Discussion

A better understanding of the molecular mechanisms underlying the development and progression of the cervical adenocarcinoma may help to identify novel targets for pharmacological intervention in this devastating disease. We have shown that factors secreted by endothelial cells increase tumor cell survival and migration *in vitro*[26]. Here, we investigated the impact of endothelial cell-initiated signaling events to the pathobiology of cervical adenocarcinomas *in vivo.*

It has been shown that conditioned medium collected from endothelial cells stimulate phosphorylation of STAT3, Akt, and ERK in head and neck squamous cell carcinomas [26]. However, it is not known whether the ability to activate these pathways was unique to endothelial cells, or if tumor cells themselves could also induce these signaling events. Several studies describe an autocrine effect of tumor cell-secreted factors on cancer progression [31-33]. Here, we demonstrated that tumor cells exposed to endothelial cell conditioned medium showed significantly higher levels of STAT3, Akt, and ERK phosphorylation than tumor cells exposed to conditioned medium collected from tumor cells. Several studies have shown that deregulation of STAT3, Akt, and ERK signaling is implicated in tumorigenesis [34-39], suggesting that aberrant activity of a network of interrelated signaling pathways, rather than a single deregulated route, contributes to carcinogenesis. We observed that while levels of total STAT3, Akt, and ERK were uniformly distributed throughout the xenograft tumors, the expression of phosphorylated STAT3, Akt, and ERK was more clustered around blood vessels. These results provide further evidence that endothelial cell-secreted factors may play a role in the activation of these pathways within the tumor microenvironment.

To our knowledge, the crosstalk between STAT3, Akt, and ERK pathways has not been studied in cervical cancer. Trying to understand the relationship between these endothelial cell-initiated signaling events on tumor cells, we exposed tumor cells to endothelial cell conditioned medium in the presence of chemical inhibitors of STAT3, Akt, and ERK pathways. Our results showed that endothelial cell-induced Akt and ERK signaling have a mutually compensatory effect, while STAT3 pathway appears to be activated independently. These results are in accordance with accumulating evidence that Akt and ERK pathways may cooperate to promote the survival of transformed cells, and are alternatively and/or coordinately expressed in several cancers, raising the possibility that a feedback loop might exist in this network [40-44].

It is well established that activation of the STAT3 signaling pathway promotes tumor growth and expression of pro-angiogenic factors [45]. We observed that blockade of endothelial cell-derived IL-6 inhibited STAT3 phosphorylation in cancer cells [26] and expression of CXCL8 (IL-8), a potent pro-angiogenic factor that is strongly correlated with tumor microvessel density [46]. Indeed, despite the fact that endothelial cells secrete many cytokines and growth factors, silencing of IL-6 with shRNA (or use of a netutralizing antibody) completely abrogated induced phosphorylation of STAT3 in tumor cells [26]. Notably, expression of IL-6 is higher in endothelial cells than in the tumor cells themselves (data not show). Here, we reported that xenograft tumors engineered with endothelial cells stably transduced with shRNA-IL-6 exhibit lower microvessel density. These results corroborate the hypothesis that IL-6 mediates a pro-angiogenic paracrine loop that plays an important role in tumor growth and angiogenesis. In other words, downregulation of IL-6 secreted by endothelial cells inhibits phosphorylation of STAT3 in tumor cells, which will then secrete less angiogenic factors (*e.g.* CXCL8) causing a decrease in tumor microvessel density and tumor growth.

Notably, tumor cells expressing phosphorylated STAT3 localized primarily adjacent to blood vessels and correlated with expression of the proliferation marker Ki67. We only analyzed Ki67 positivity adjacent to blood vessels in both groups to eliminate possible differences due to hypoxia. Expression of Ki67 in tumor cells and tumor microvessel density were lower in tumors vascularized with IL-6-silenced endothelial cells. Early studies have shown that Bcl-2 is upregulated in tumor-associated endothelial cells, that upregulation of Bcl-2 in microvascular endothelial cells accelerates tumor growth, and that endothelial cells overexpressing Bcl-2 secrete higher levels of IL-6 than vector control cells [25-28] These findings, along with the results presented here, begin to provide a possible mechanism for the impact of endothelial cell-derived IL-6 on tumor growth.

## Conclusion

Targeted disruption of the vascular endothelium has been proposed by Dr. Folkman four decades ago and has shown efficacy in some tumor types [11-13,47]. However, this approach results in hypoxic, nutrient-deprived tumor microenvironments that can be associated with enhanced motility of tumor cells and development of evasive resistance to therapy [48]. Here, we showed that specific blockade of the endothelial cell-tumor cell crosstalk (*e.g.* IL-6) is sufficient to inhibit tumor growth. These results suggest that cervical cancer patients might benefit from the therapeutic blockade of key signaling events that regulate the crosstalk between endothelial cells and tumor cells.

## Competing interests

The authors have no competing of interest to declare.

## Authors’ contributions

KGN participated in the design of the study, carried out the *in vitro* and *in vivo* experiments and drafted the manuscript. KAW and MSC participated in the mouse experiments, and ZZ participated in the generation of the stable cell lines. JM and TED helped to draft the manuscript and provided clinic/pathologic expertise for this work. JEN conceived the study, participated in its design and coordination and helped to draft the manuscript. All authors read and approved the final manuscript.

## Pre-publication history

The pre-publication history for this paper can be accessed here:

http://www.biomedcentral.com/1471-2407/14/99/prepub

## Supplementary Material

Additional file 1: Figure S1 Blockade of endothelial cell-induced STAT3 phosphorylation in tumor cells does not affect Akt and ERK pathways, whereas inhibition of Akt or ERK has a compensatory mechanism. HeLa cells were serumstarved overnight and exposed to A, HDMEC conditioned medium (CM) or unconditioned medium (EBM) for the indicated time points. In addition, HeLa cells were pre-incubated for 1 to 2 hours with B, 20 μM Stattic, C, 20 μM LY294002, or D, 20 μM U0126, and then exposed to HDMEC CM or EBM for the indicated time points. Phosphorylated and total STAT3, Akt, and ERK levels were determined by Western blot. Figure S2. IL-6 potently activates STAT3 signaling in cervical adenocarcinoma cells. HeLa cells were serum-starved overnight and exposed to 20 ng/ml rhIL-6 for the indicated time points. Phosphorylated and total levels of STAT3, Akt, and ERK were determined by Western blots. A, HeLa cells exposed to rhIL-6. HeLa cells pre-incubated for 1 to 2 hours with B, 20 μM Stattic; C, 20 μM LY294002; or D, 20 μM U0126, and then exposed to rhIL-6 for the indicated time points. Click here for file
