# Supplementary material for: Endothelial cell-derived interleukin-6 regulates tumor growth
Source: BMC Cancer. 2014 Feb 17;14:99. doi: 10.1186/1471-2407-14-99 (PMC4016552; doi:10.1186/1471-2407-14-99)
Supplement: Additional file 1: Figure S1 — Blockade of endothelial cell-induced STAT3 phosphorylation in tumor cells does not affect Akt and ERK pathways, whereas inhibition of Akt or ERK has a compensatory mechanism. HeLa cells were serumstarved overnight and exposed to A, HDMEC conditioned medium (CM) or unconditioned medium (EBM) for the indicated time points. In addition, HeLa cells were pre-incubated for 1 to 2 hours with B, 20 μM Stattic, C, 20 μM LY294002, or D, 20 μM U0126, and then exposed to HDMEC CM or EBM for the indicated time points. Phosphorylated and total STAT3, Akt, and ERK levels were determined by Western blot. Figure S2. IL-6 potently activates STAT3 signaling in cervical adenocarcinoma cells. HeLa cells were serum-starved overnight and exposed to 20 ng/ml rhIL-6 for the indicated time points. Phosphorylated and total levels of STAT3, Akt, and ERK were determined by Western blots. A, HeLa cells exposed to rhIL-6. HeLa cells pre-incubated for 1 to 2 hours with B, 20 μM Stattic; C, 20 μM LY294002; or D, 20 μM U0126, and then exposed to rhIL-6 for the indicated time points. [file 1471-2407-14-99-S1.pdf]

Supplementary Figure Legends

Supplementary Figure S1. Blockade of endothelial cell-induced STAT3 phosphorylation in tumor cells does not affect Akt and ERK pathways, whereas inhibition of Akt or ERK has a compensatory mechanism. HeLa cells were serum-starved overnight and exposed to *A,* HDMEC conditioned medium (CM) or unconditioned medium (EBM) for the indicated time points. In addition, HeLa cells were pre-incubated for 1 to 2 hours with *B,* 20 μM Stattic, *C,* 20 μM LY294002, or *D,* 20 μM U0126, and then exposed to HDMEC CM or EBM for the indicated time points. Phosphorylated and total STAT3, Akt, and ERK levels were determined by Western blot.

Supplementary Figure S2. IL-6 potently activates STAT3 signaling in cervical adenocarcinoma cells. HeLa cells were serum-starved overnight and exposed to 20 ng/ml rhIL-6 for the indicated time points. Phosphorylated and total levels of STAT3, Akt, and ERK were determined by Western blots. *A*, HeLa cells exposed to rhIL-6. HeLa cells pre-incubated for 1 to 2 hours with *B,* 20 μM Stattic; *C,* 20 μM LY294002; or *D,* 20 μM U0126, and then exposed to rhIL-6 for the indicated time points.

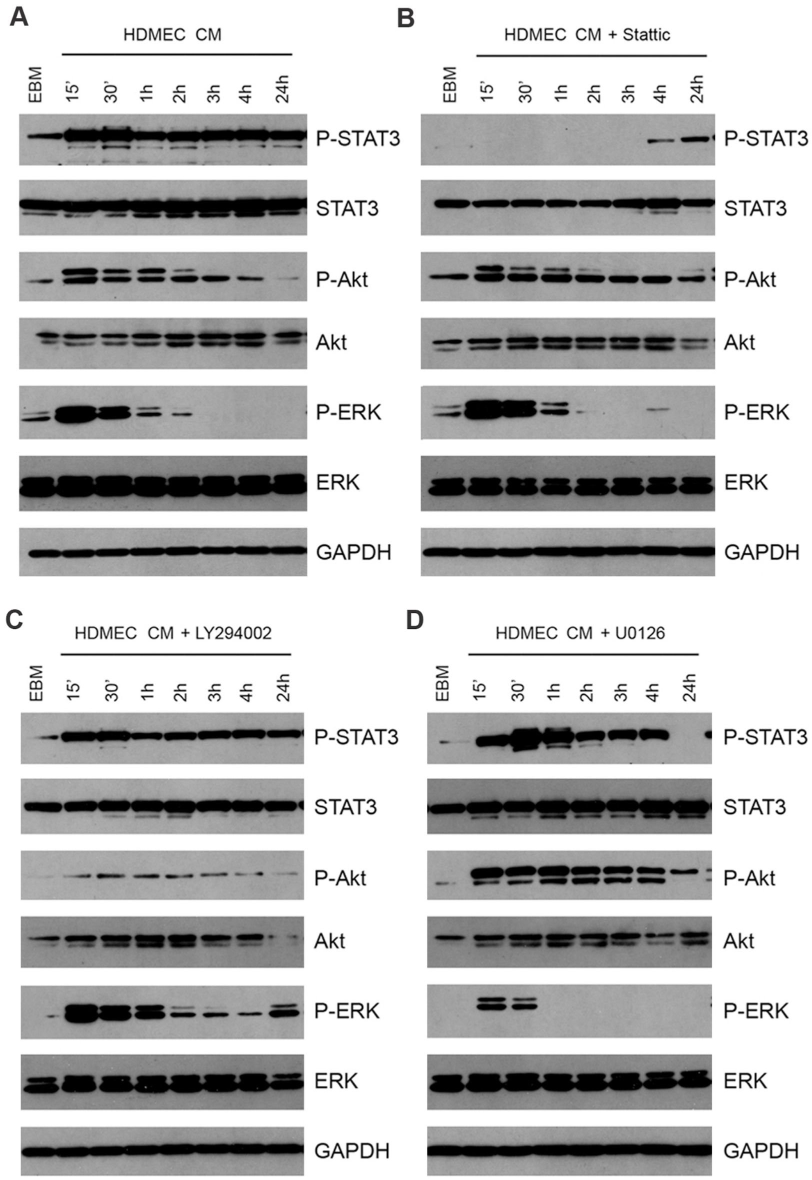

Supplementary Figure S1 - Neiva et al.

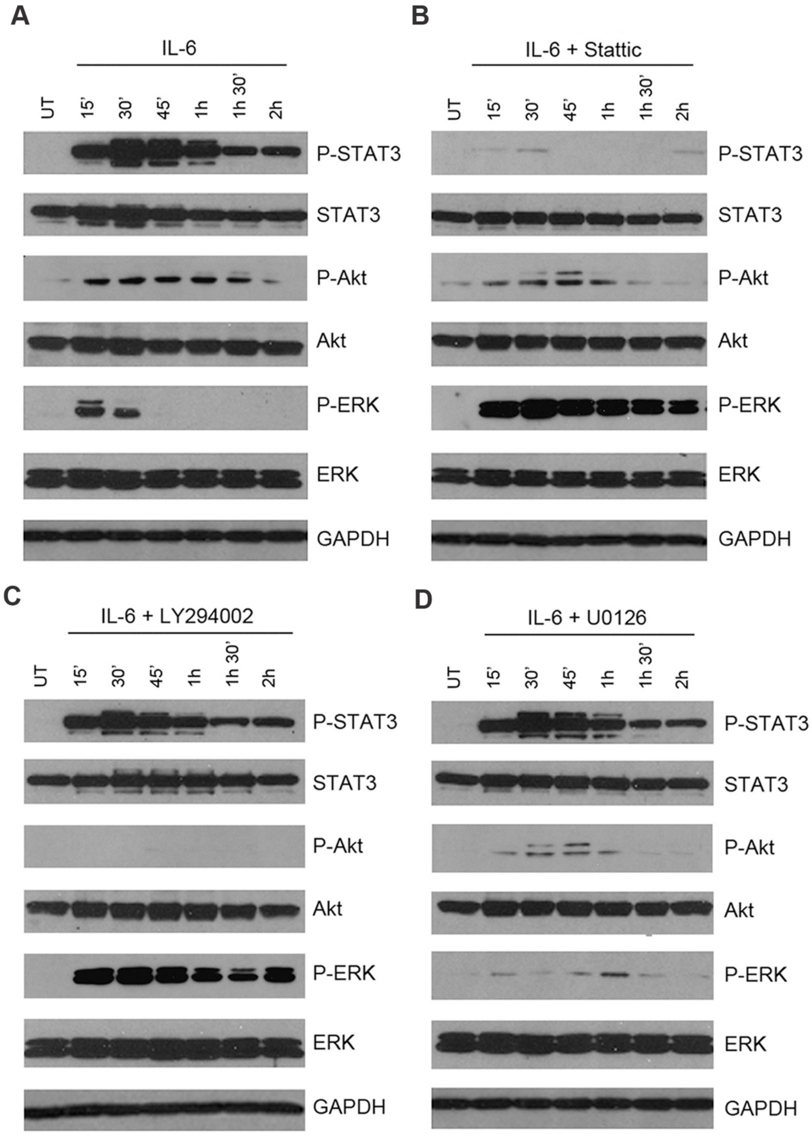

Supplementary Figure S2 - Neiva et al.
